# Supplementary figures and images for: Divergent role of Mitochondrial Amidoxime Reducing Component 1 (MARC1) in human and mouse
Source: PLoS Genet. 2024 Mar 4;20(3):e1011179. doi: 10.1371/journal.pgen.1011179 (PMC10939284; doi:10.1371/journal.pgen.1011179)

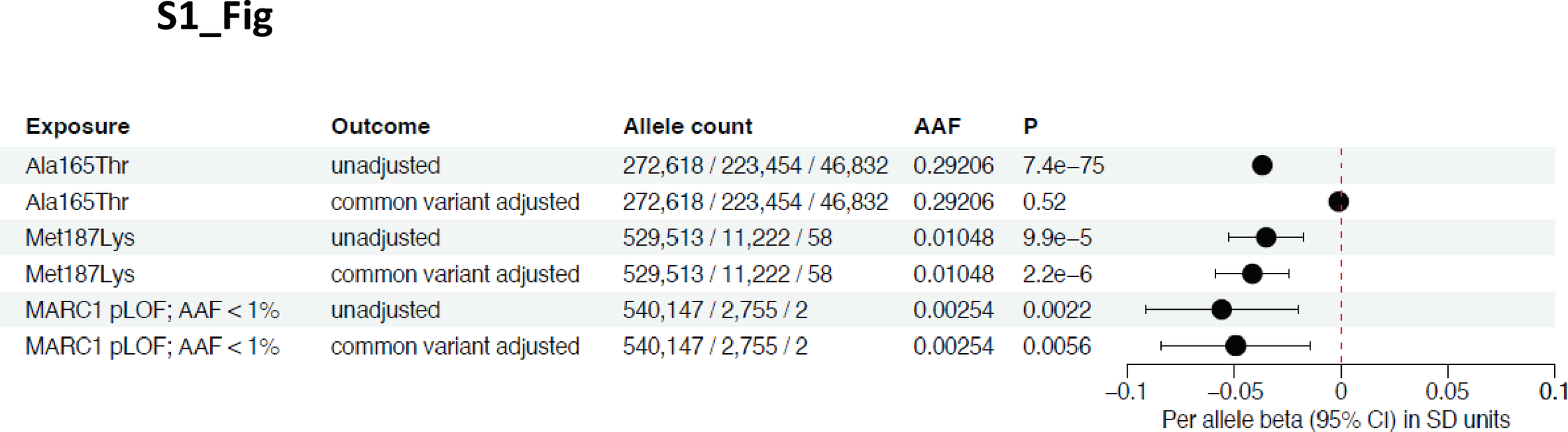

Supplement: S1 Fig — Rare variant adjusted by common variants. ALT (U/L); AAF—alternate allele frequency. (TIF) [file pgen.1011179.s001.tif]

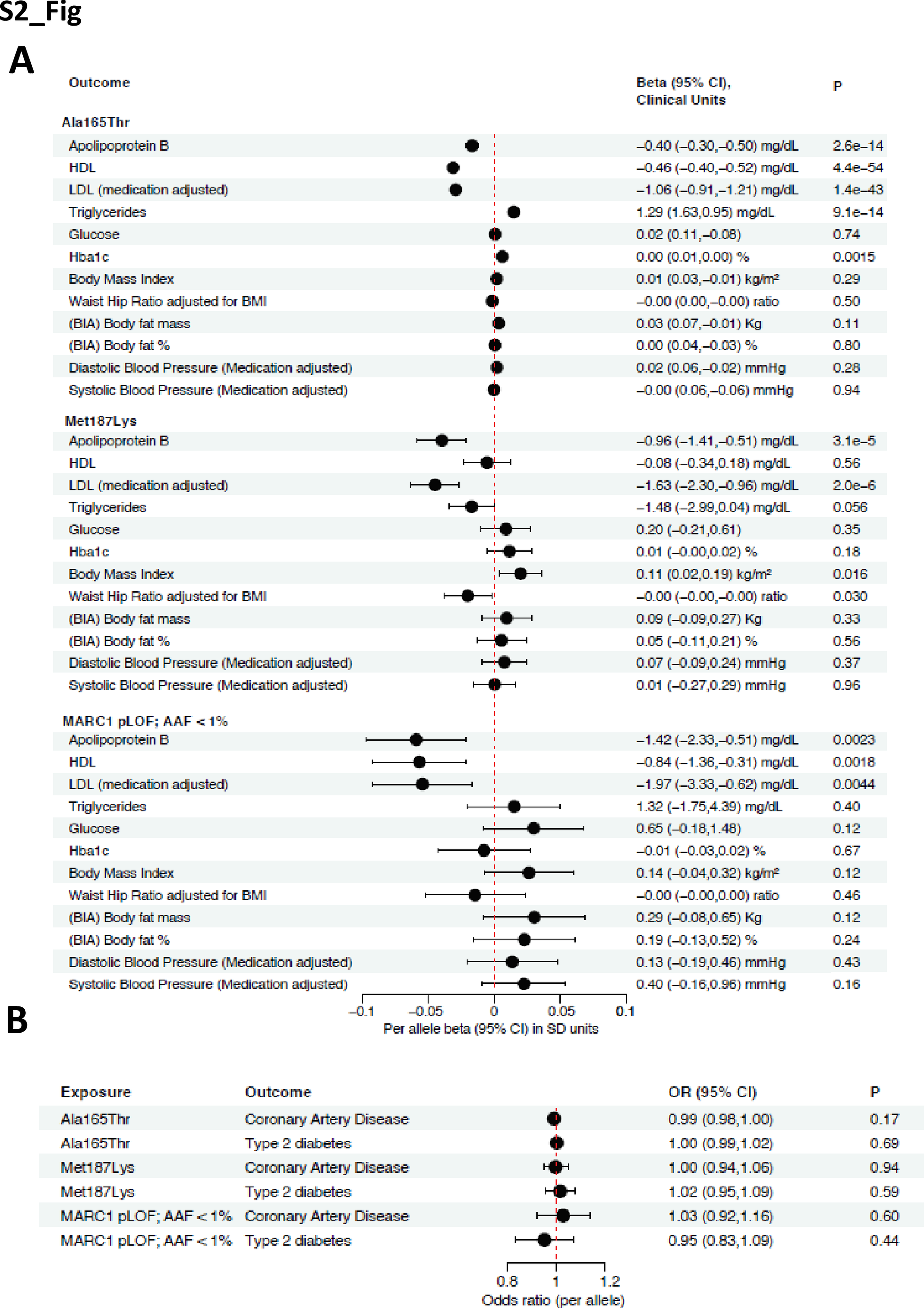

Supplement: S2 Fig — (A) Serum lipids, glucose, body mass index and blood pressure, (B) type 2 diabetes and coronary artery disease. (TIF) [file pgen.1011179.s002.tif]

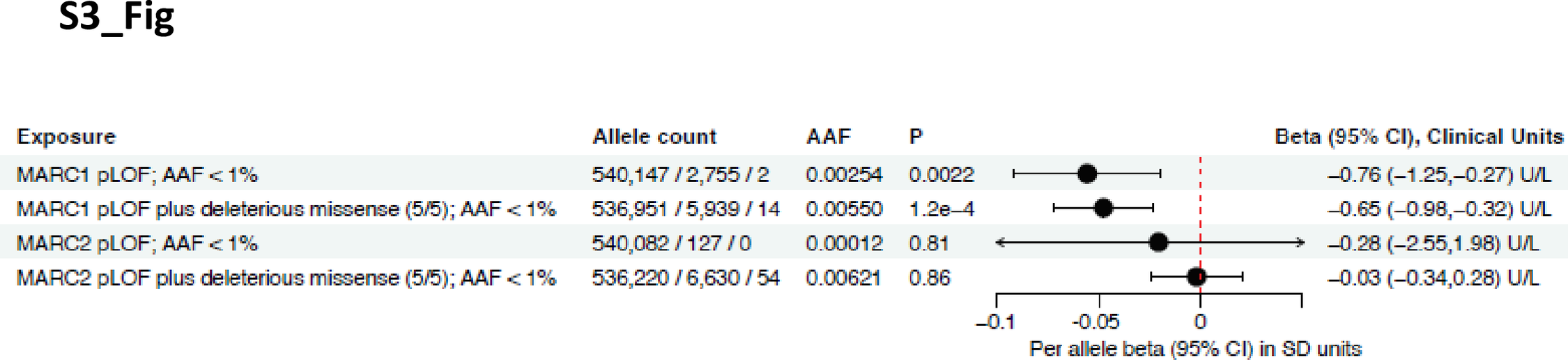

Supplement: S3 Fig — AAF—alternate allele frequency. (TIF) [file pgen.1011179.s003.tif]

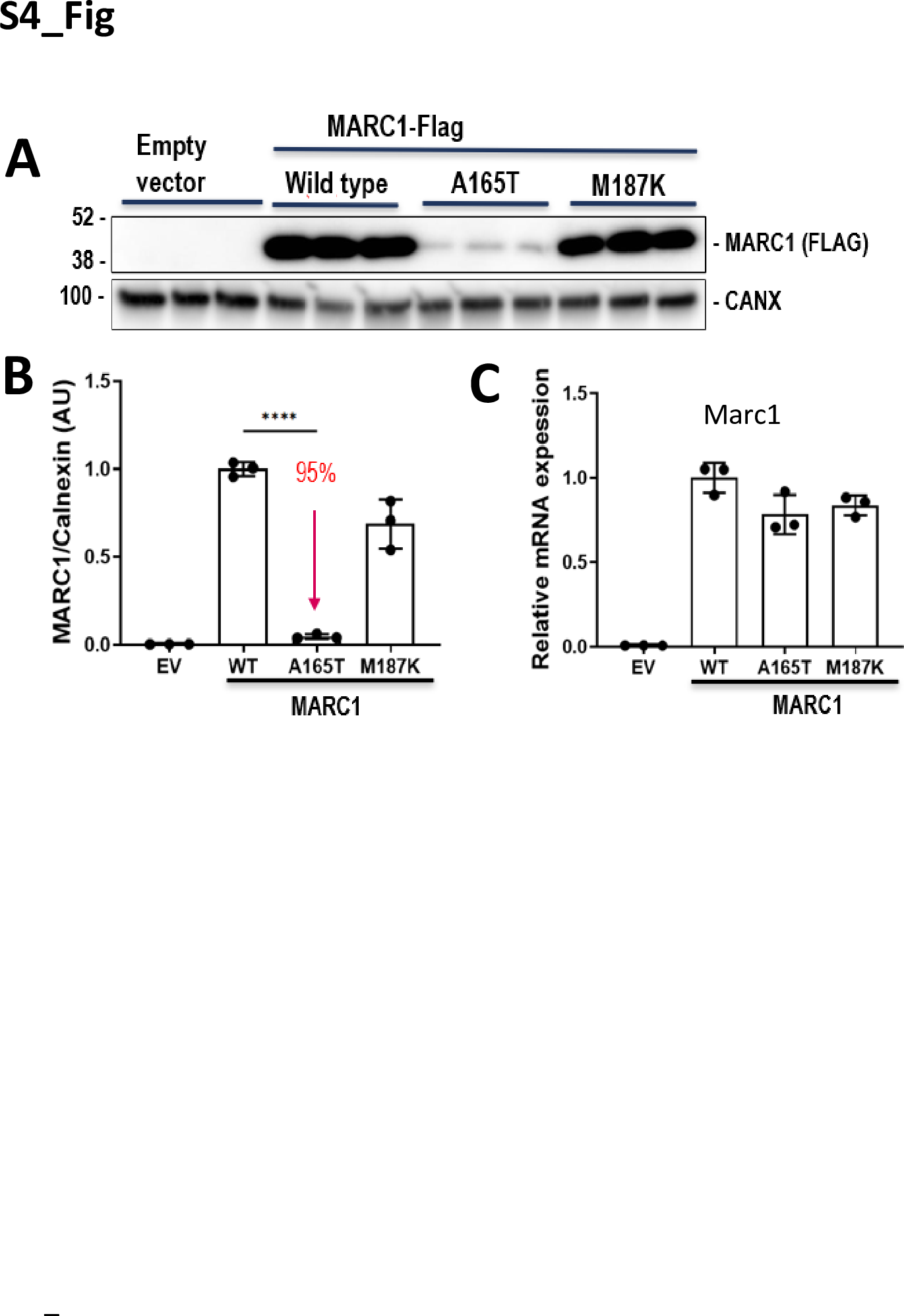

Supplement: S4 Fig — (A) Plasmids with empty vector (Vector), wild-type and common variants of human MARC1 (CMV promoter, C terminal 1x Flag tag) were expressed in HepG2 cells. 48 hours after transfection, cells were split into two parts. First part of cells were solubilized in RIPA buffer, proteins separated on SDS-PAGE (4–20%) gels and blotted with corresponding antibodies, (B) immunoblot quantitation was performed. (C) Second part of cells was used to measure MARC1 mRNA levels using Real-Time PCR. Mean ± s.e.m. are shown in all graphs, ****p<0.0001 (TIF) [file pgen.1011179.s004.tif]

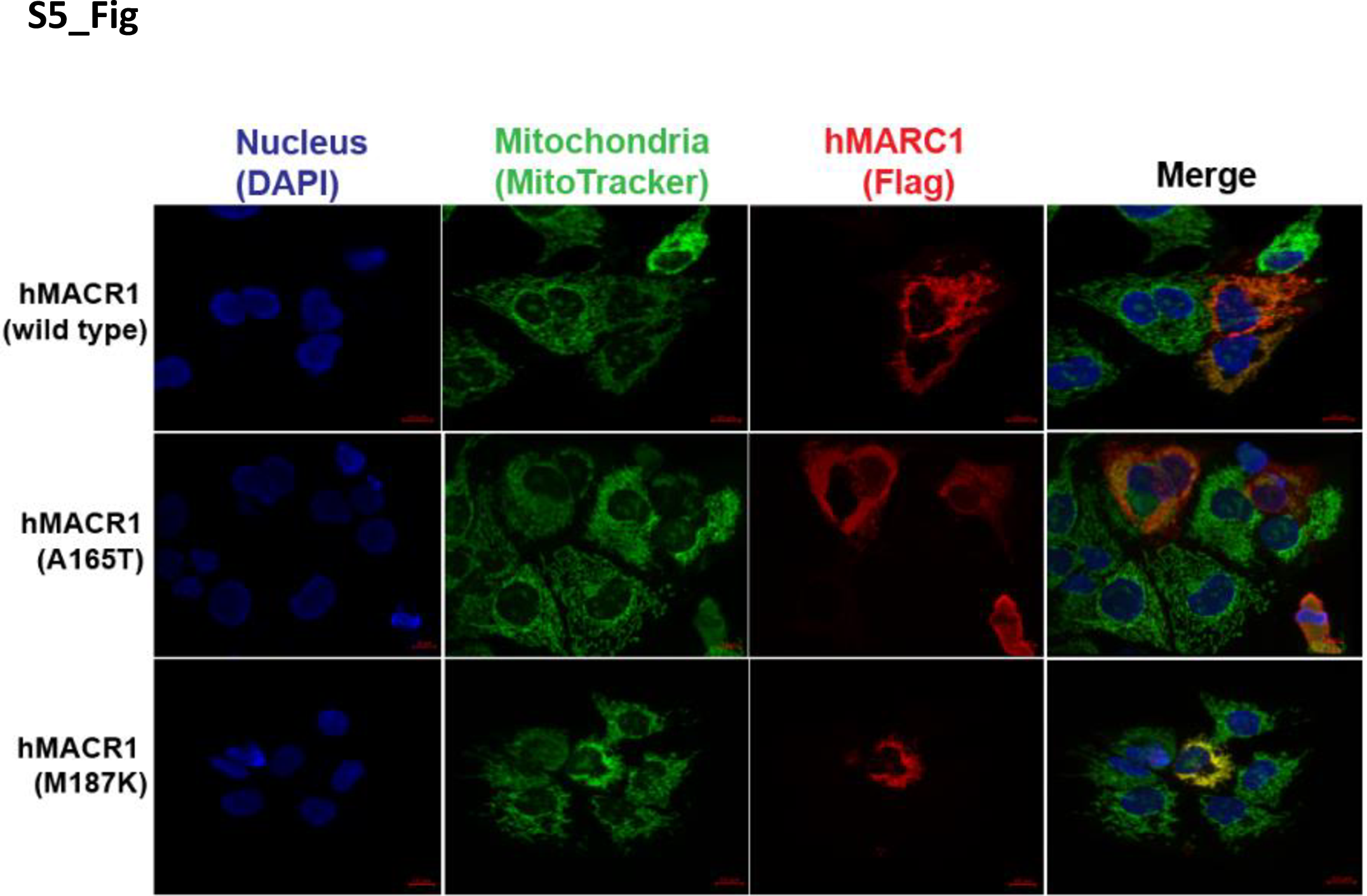

Supplement: S5 Fig — HuH-7 cells were transfected with plasmids containing wild type or 2 common genetic variants (p.A165T and p.M187K) of human MARC1 (with C terminal Flag tag and gene expression driven under CVM promoter). 48 hours after transfection cells were incubated with Mitotracker Orange CMTMRos staining (Thermo Fisher) solution in serum free media for 20 minutes, cells were washed, fixed, permeabilized and blocked as described in Methods. After additional washing, cells were incubated with Flag antibody (Sigma) for 1 hour, washed and incubated with Alexa Fluor 647 conjugated secondary antibody. After final wash, cells were incubated in mounting medium with DAPI (Ibidi) and imaged with Leica confocal microscope. The experiment was repeated once with the equivalent results. (TIF) [file pgen.1011179.s005.tif]

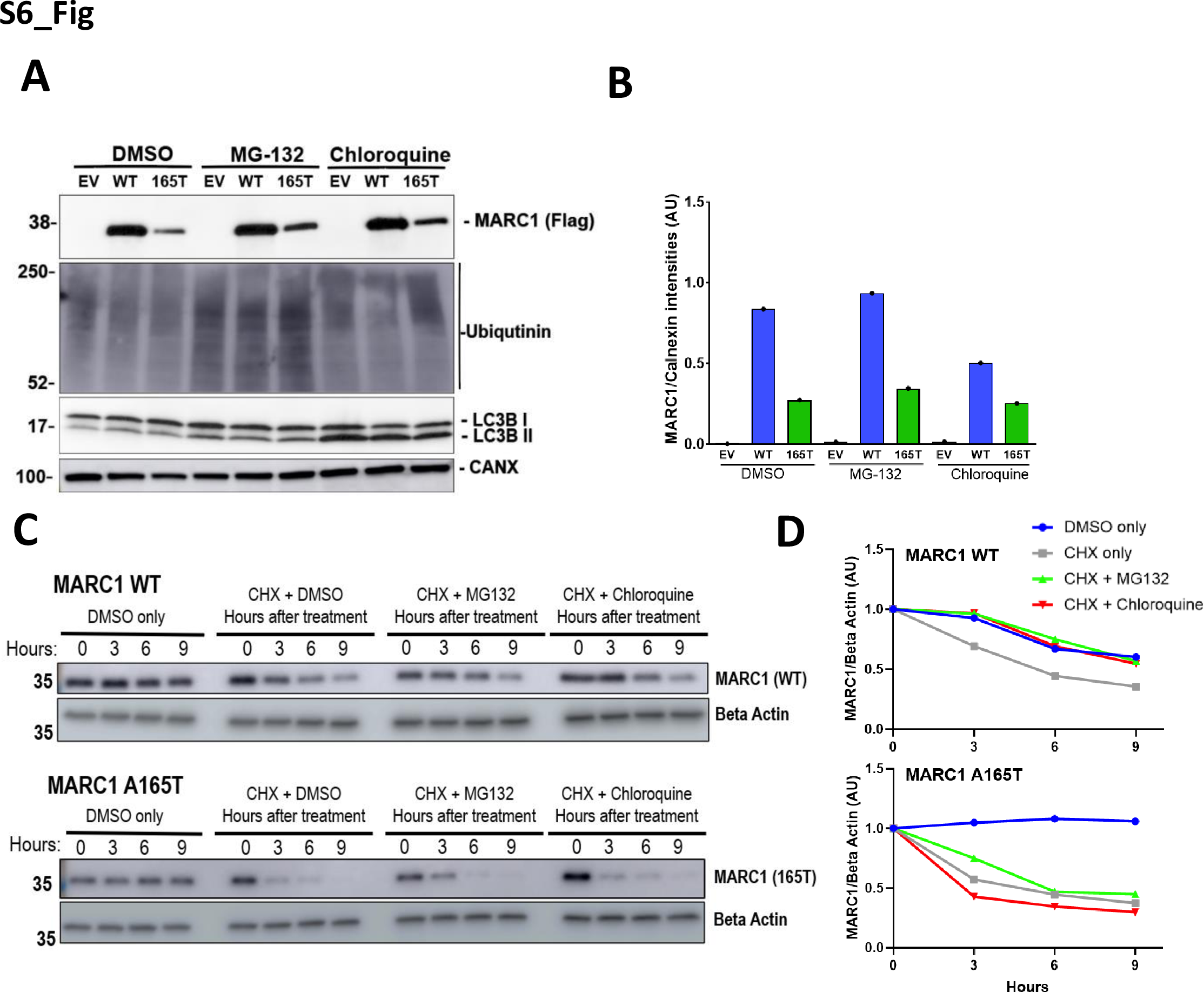

Supplement: S6 Fig — (A) Plasmids encoding empty vector (EV), wild-type (WT) and common variants (165T) of full length human MARC1 (with C terminal FLAG tag) were transfected into HuH-7 cells. 48 hours after transfection, cell media containing DMSO only (vehicle) or 10 μM MG-132 in DMSO (proteasomal inhibitor) or 10ug/ml chloroquine in water (lysosomal inhibitor) plus DMSO separately were added and cells were incubated for additional 8 hours. After that, cells were washed, collected and solubilized in RIPA buffer, proteins separated on SDS-PAGE (4–20%) gels and blotted with corresponding primary antibodies. (B) Protein densitometry was performed on blots (A) and ratio between MARC1 variants and Calnexin were calculated. (C) Cells were prepared and transfected with WT or A165T variant human MARC1 similar as in (A), 48 h after transfection cells were incubated with DMSO or cycloheximide (CHX; 300 μg/ml) in DMSO or CHX (300 μg/ml) plus 10 μM MG-132 in DMSO or CHX (300 μg/ml) plus 10ug/ml chloroquine in DMSO for 0, 3, 6 and 9 hours. After incubation cells were collected and processed as in (A). (D) Protein densitometry was performed on (C) blots and ratios between MARC1 variants and β-actin were calculated, the value of 0 timepoint for each treatment condition were set as 1 and the relative changes in the protein levels were calculated over time. Ubiquitin was used as proteasomal inhibitor control, LC3B-I/II as lysosomal inhibitor control. CANX (calnexin) and β-actin–as protein loading controls. The experiments was repeated once with the same results. (TIF) [file pgen.1011179.s006.tif]

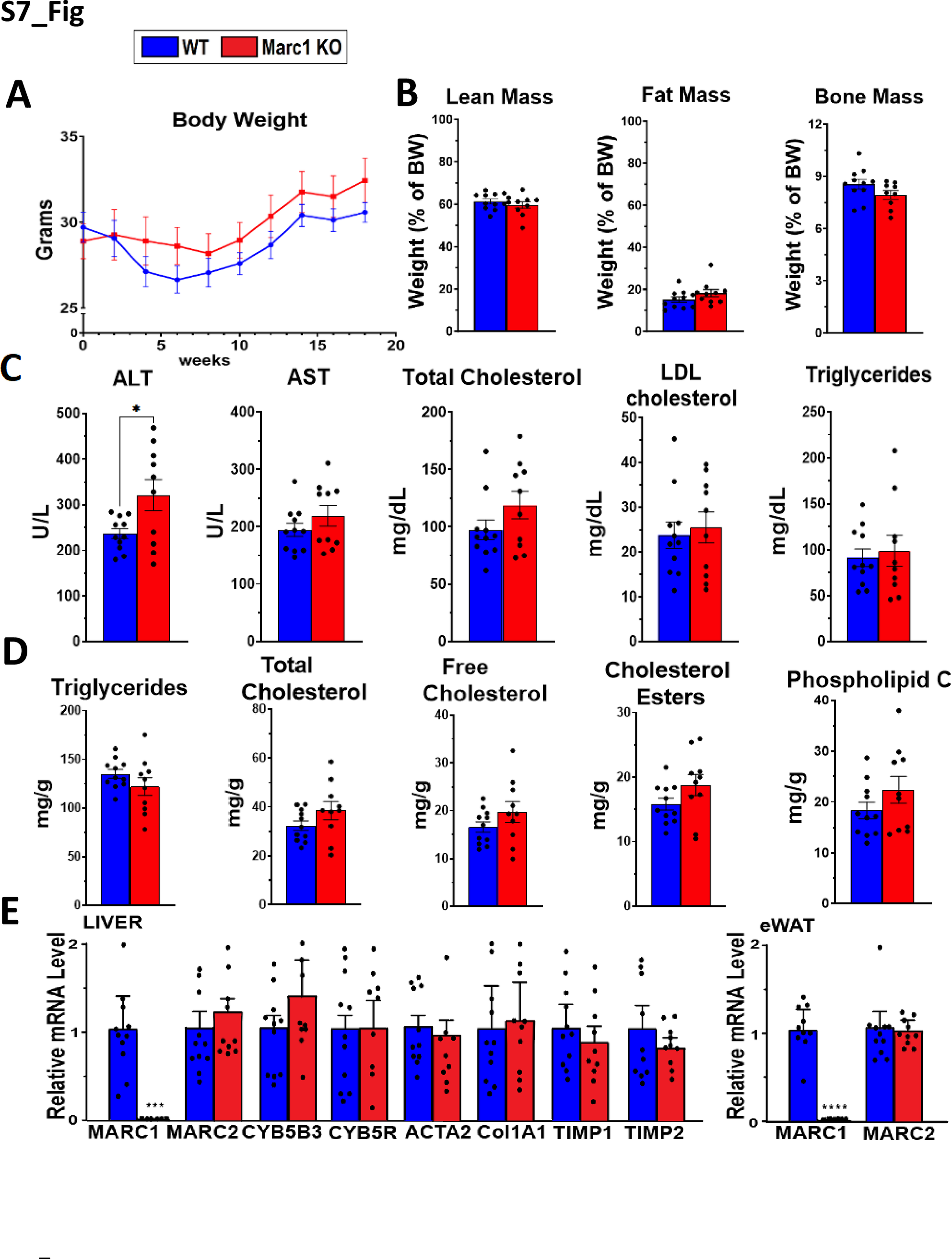

Supplement: S7 Fig — Nine weeks old male mice (11 WT and 10 Marc1 KO) were fed with CDAA-HFD for 18 weeks. (A) Body mass gain was measured every 2 weeks, (B) body composition (Micro CT) of mice were assessed at week 6 of experiment. (C) Serum, (D) liver lipids, (E) selected liver gene expression (mRNA by qRT-PCR) were performed at end of experiment. Each point represents individual mouse, mean ± s.e.m. are shown in all graphs, *p<0.05, ***p<0.001, ****p<0.0001. (TIF) [file pgen.1011179.s007.tif]

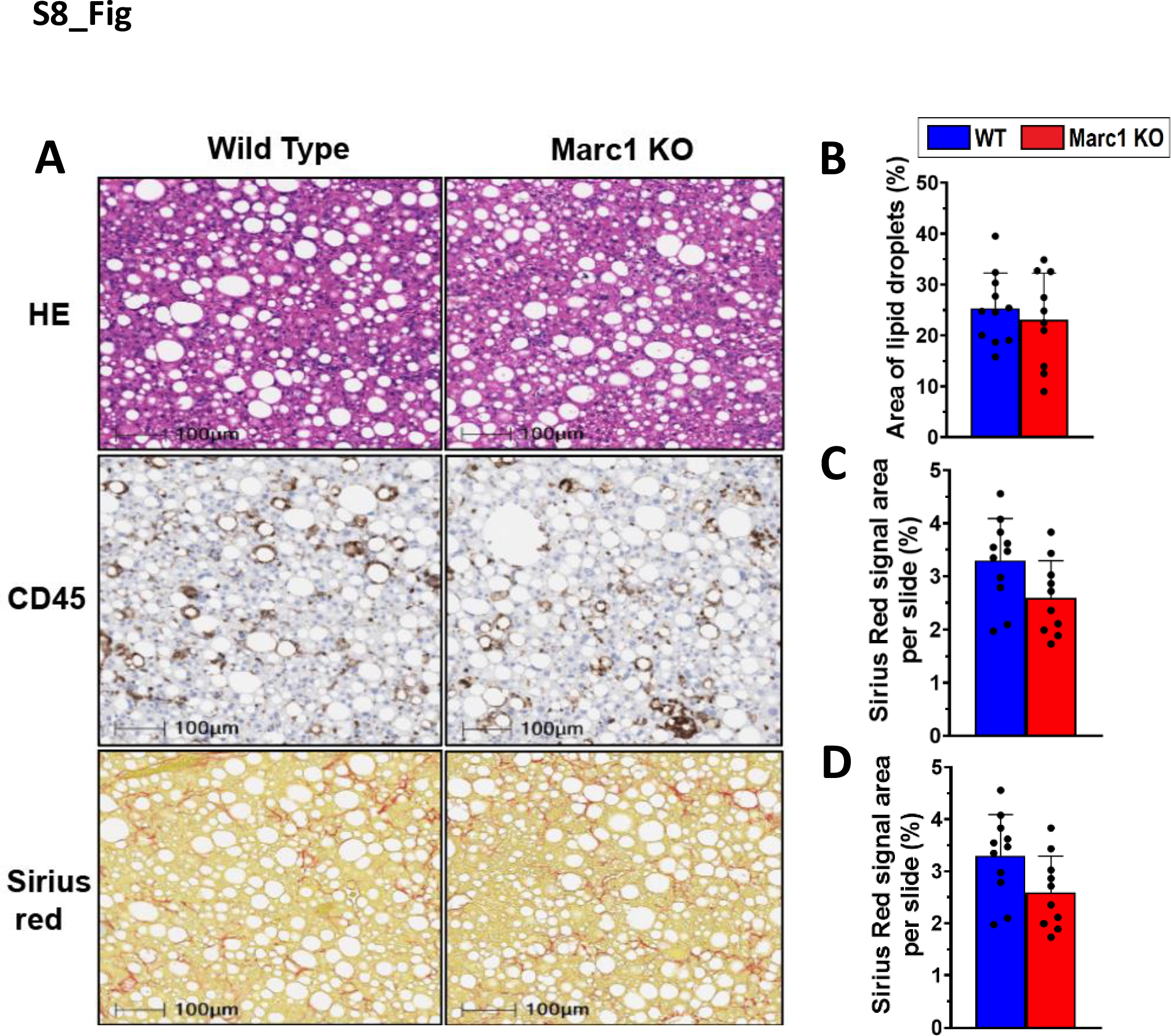

Supplement: S8 Fig — (A) Liver samples were collected from mice described in S7 Fig at end of the experiment. Fixed liver sections were stained with hematoxylin/eosin (HE), CD45 and Sirius Red staining. (B) Lipid droplet area, (C) CD45 positive cell area and (D) fibrosis area were measured from each animal liver section. Slides were scanned on Aperio AT2 scanners (Leica Biosystems) with 10X magnification. Each point represents individual mouse, mean ± s.e.m. are shown in all graphs. (TIF) [file pgen.1011179.s008.tif]

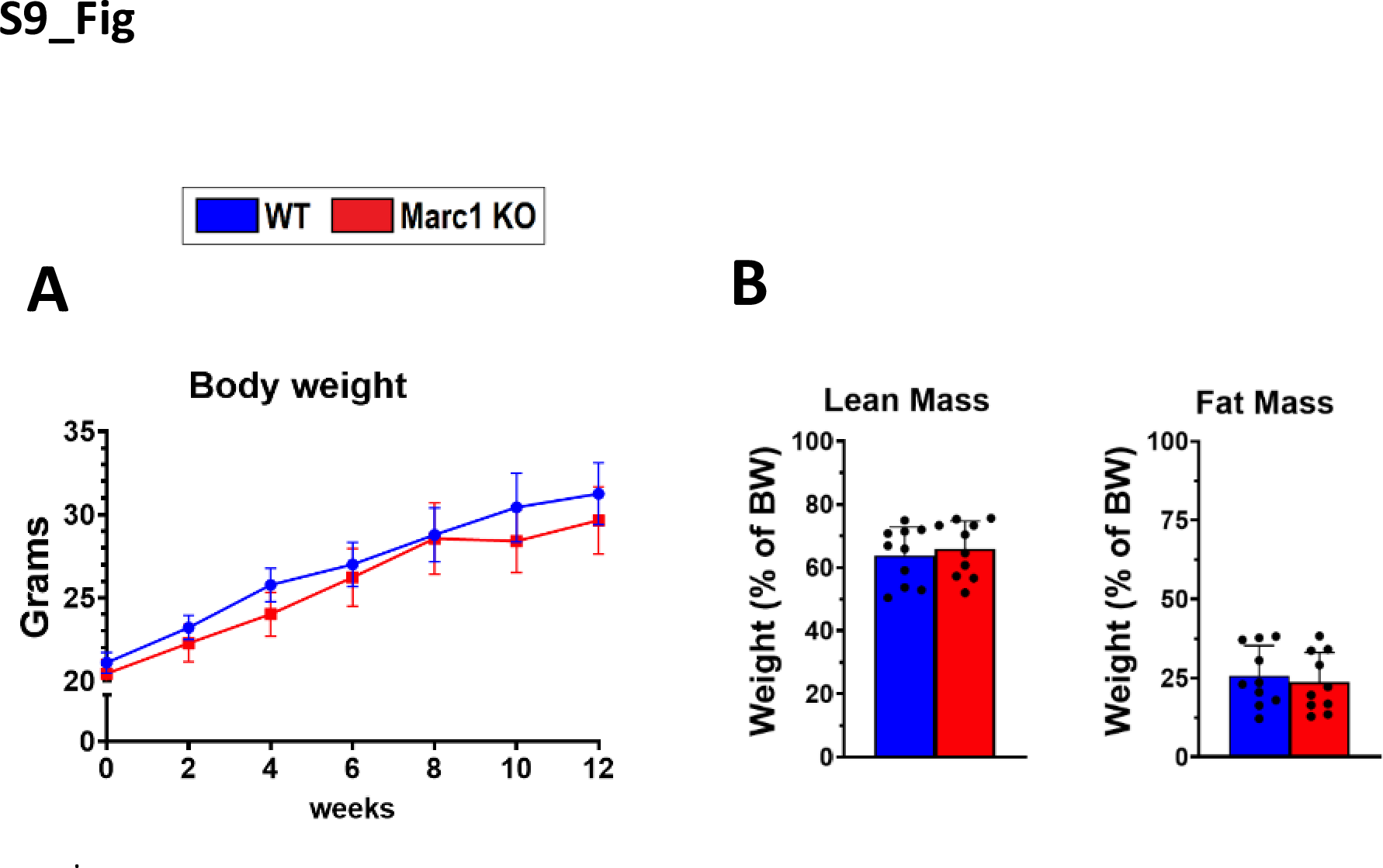

Supplement: S9 Fig — (A) 7–10 weeks old female mice were fed with HFHFD for 12 weeks. Body weight was measured every 2 weeks, (B) body composition (MRI) in mice were assessed at week 13 of experiment. Each point represents individual mouse, mean ± s.e.m. are shown in all graphs (TIF) [file pgen.1011179.s009.tif]

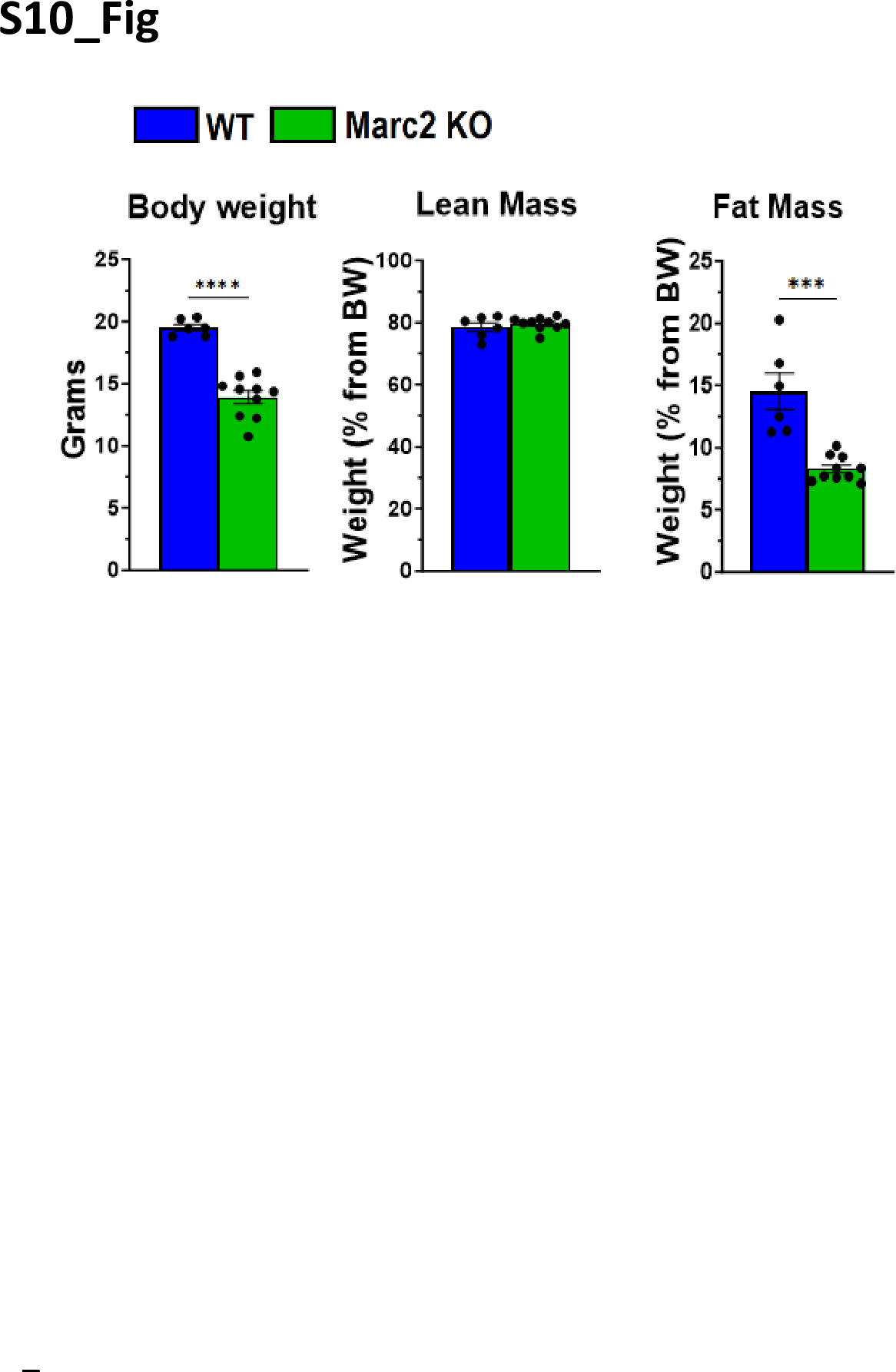

Supplement: S10 Fig — Wild type and Marc2 homozygous female mice (n = 5–9), fed chow ad lib body mass composition (Micro CT) were estimated at age 6–7 weeks. Each point represents individual mouse, mean ± s.e.m. are shown in all graphs, ***p<0.001, ****p<0.0001. (TIF) [file pgen.1011179.s010.tif]

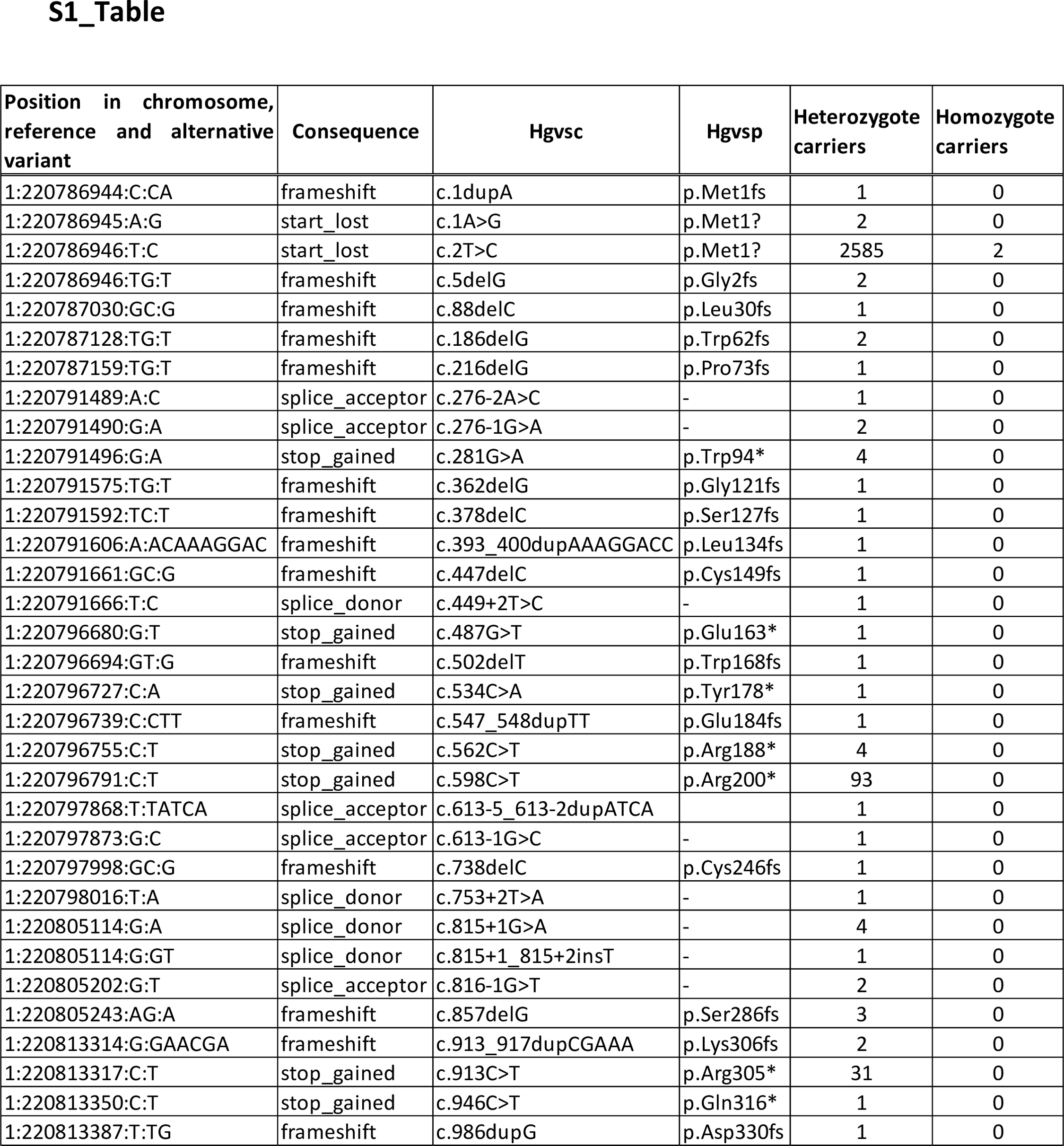

Supplement: S1 Table — Hgvsc—Human Genome Variation Society DNA variant abbreviation, Hgvsp—Human Genome Variation Society coding protein abbreviation, Met1?–skip of first Methionine of protein. (TIF) [file pgen.1011179.s011.tif]

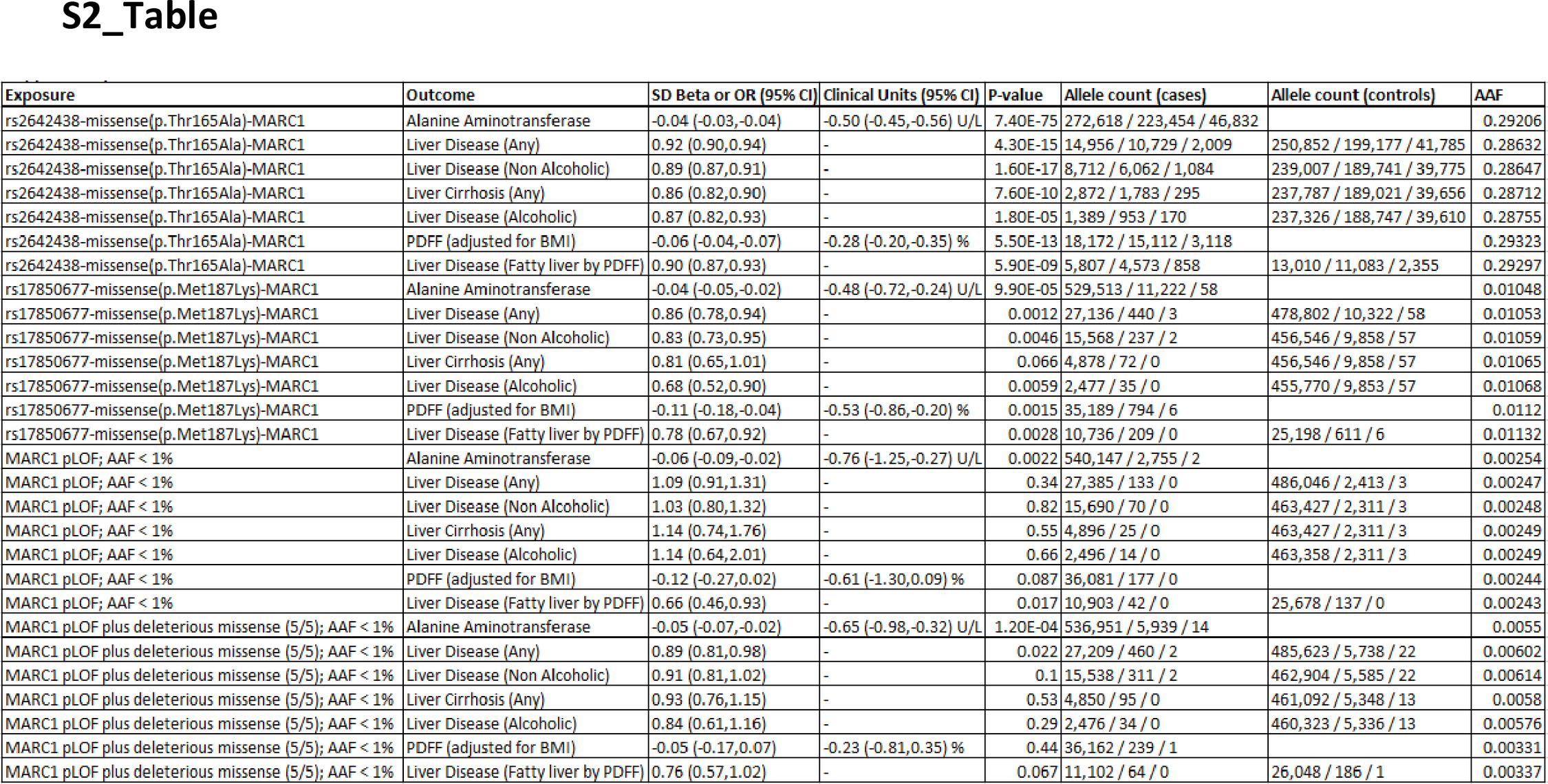

Supplement: S2 Table — AAF—alternate allele frequency, PDFF-MRI—proton density fat fraction by magnetic resonance imaging. (TIF) [file pgen.1011179.s012.tif]

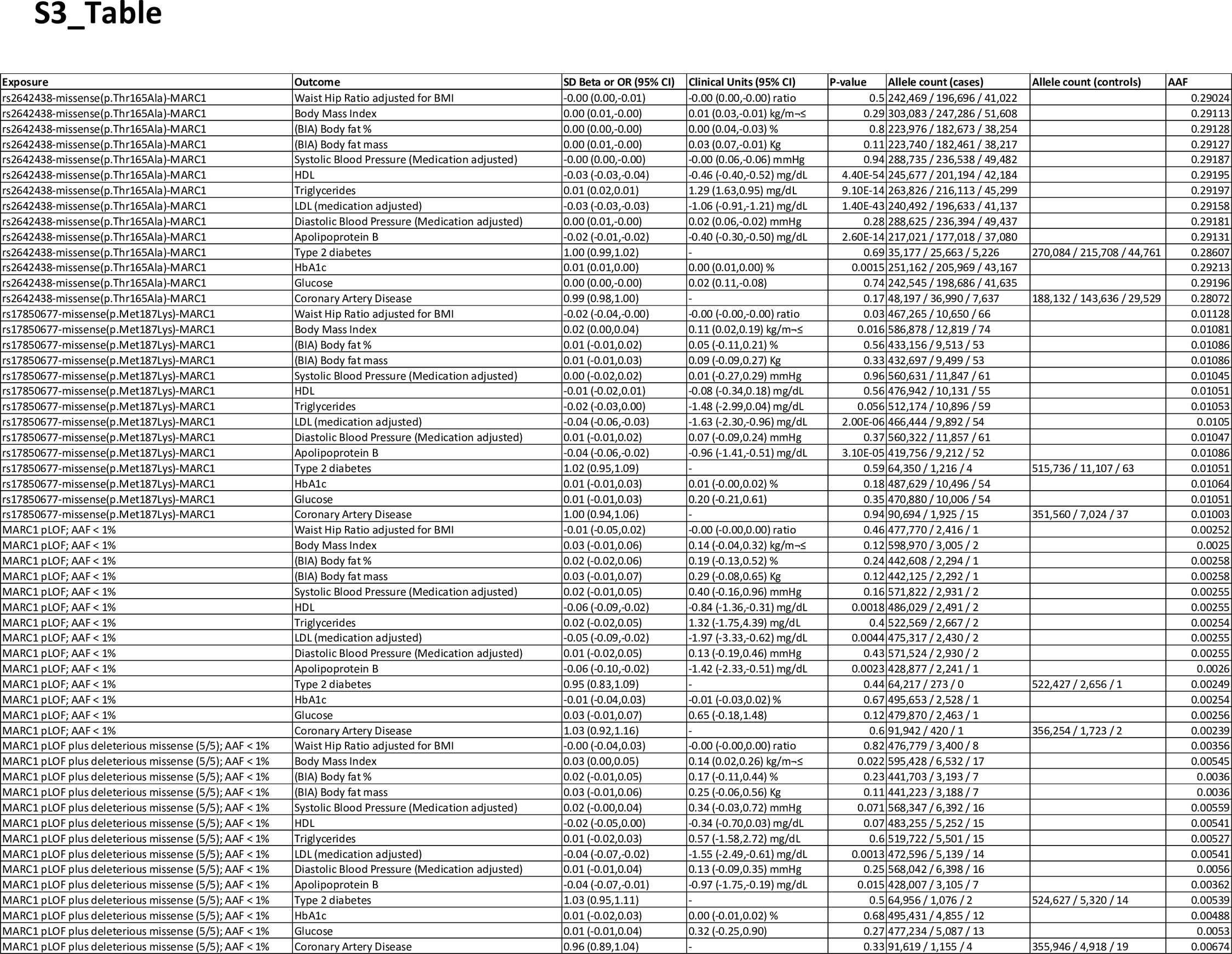

Supplement: S3 Table — AAF—alternate allele frequency. (TIF) [file pgen.1011179.s013.tif]

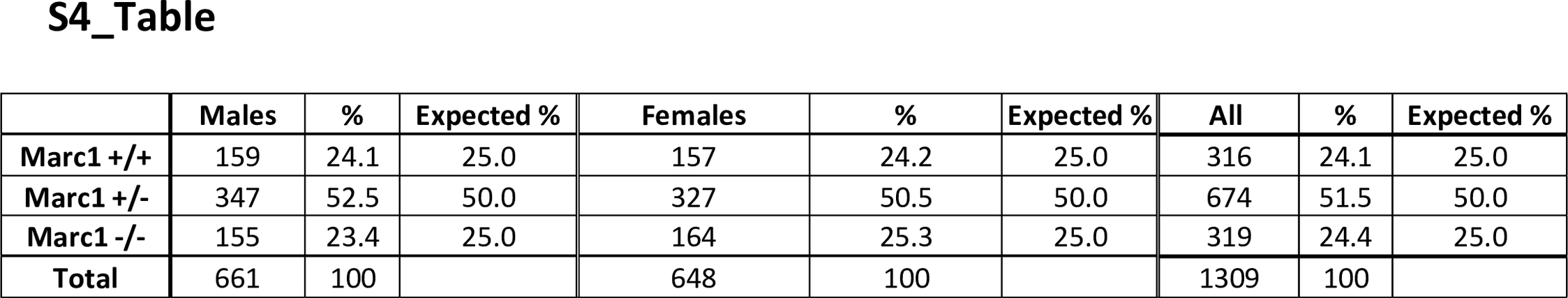

Supplement: S4 Table — Genotypes of pups from Marc1 heterozygous knockout mice breeding (F2-F4) were detected, results grouped by sex and genotype. (TIF) [file pgen.1011179.s014.tif]

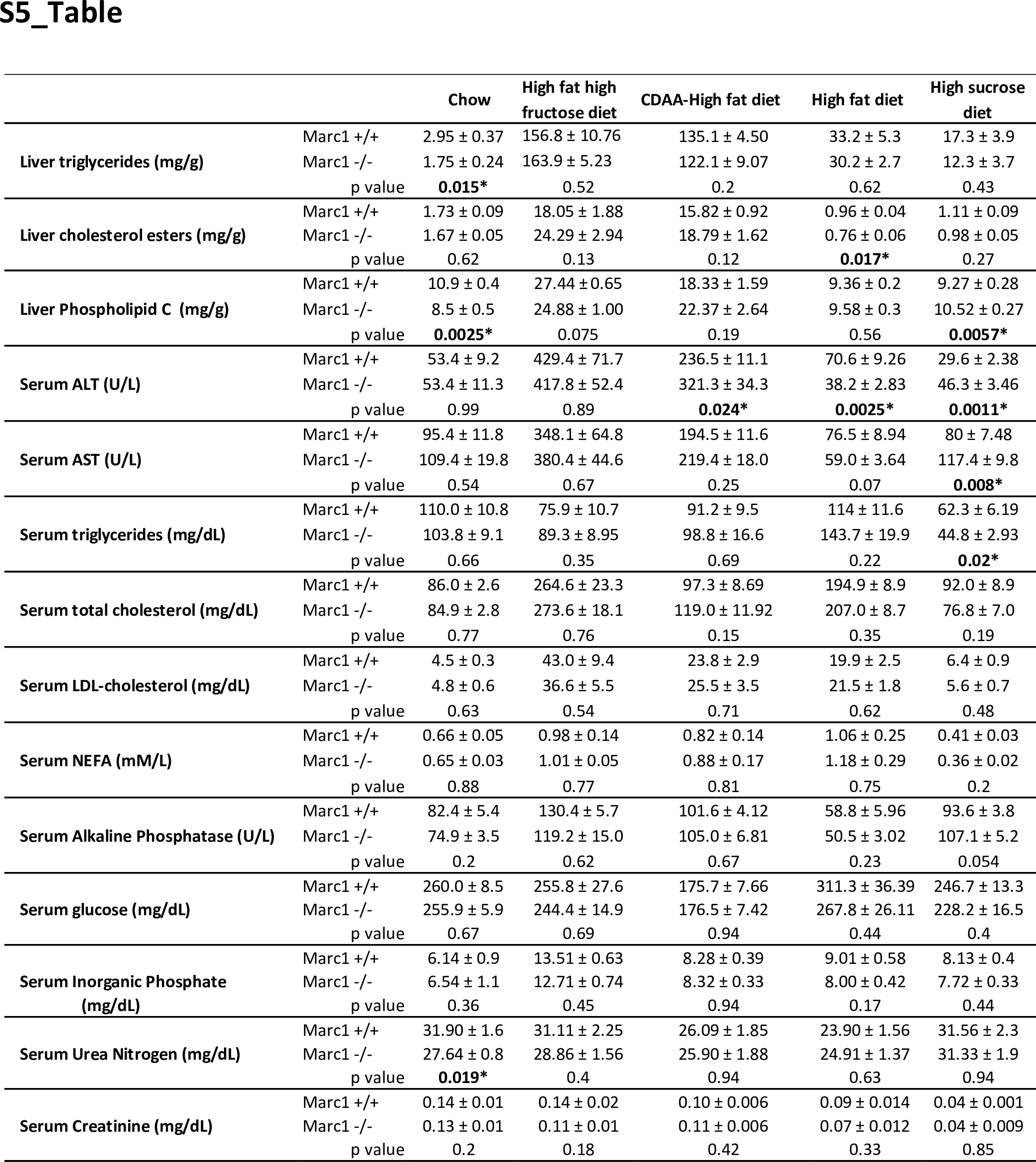

Supplement: S5 Table — Mice fed with chow diet were sacrificed at age 13 weeks. For other diets—8–10 weeks old WT and Marc1−/− male mice were fed with HFHFD for 35 weeks or CDAA-HFD for 18 weeks or high fat diet (HFD) for 11 weeks or high sucrose diet (HSD) for 9 weeks, respectively. At the end of experiments mice were sacrificed, liver and serum collected and indicated measurements done as described in Methods. Each group contains 7–12 mice. All mice were sacrificed after ad lib feeding state (non-fasted). Mean ± s.e.m. are shown for all measurements. In bold–all measurement with *p<0.05. (TIF) [file pgen.1011179.s015.tif]

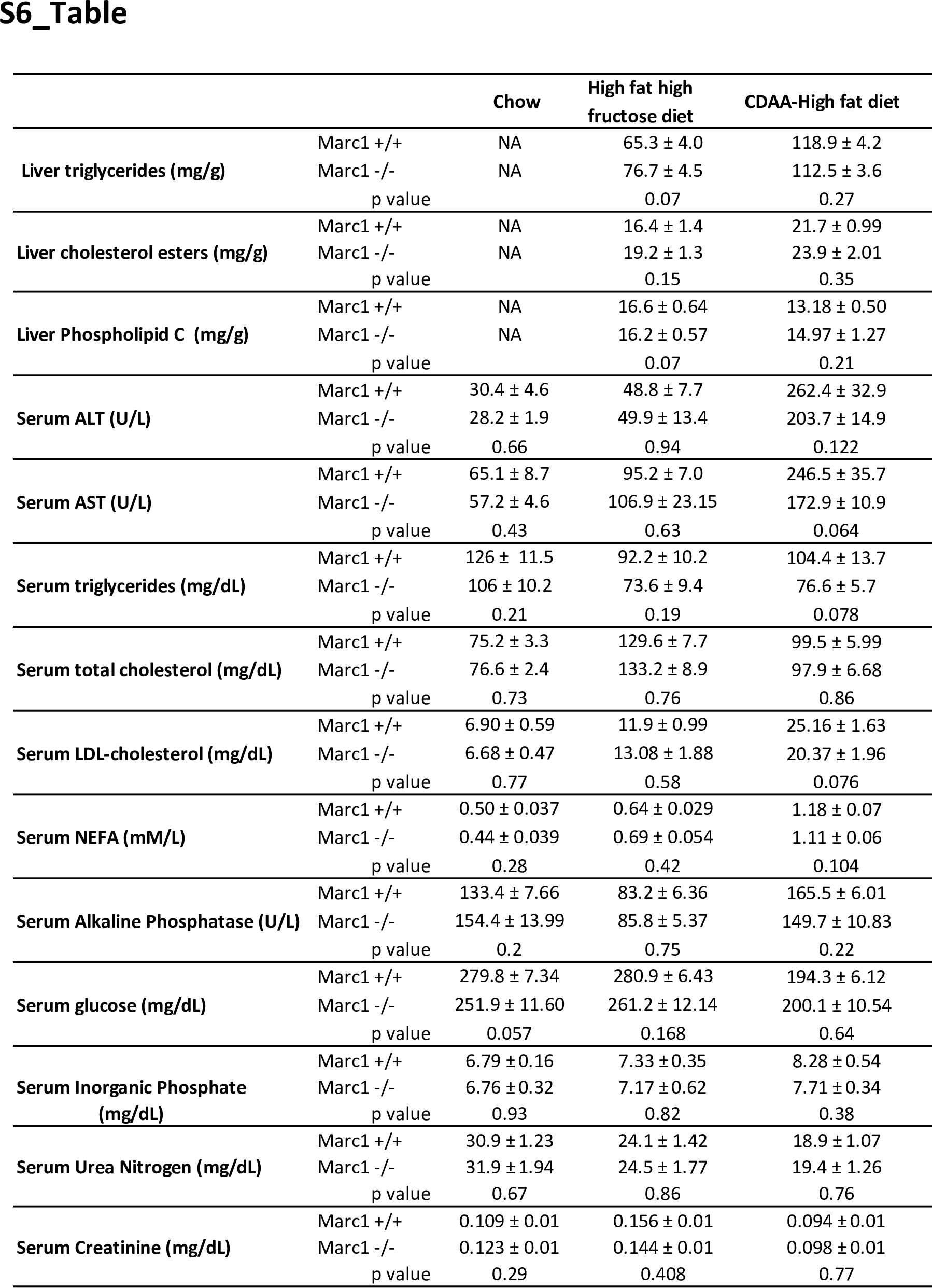

Supplement: S6 Table — Serum was collected from chow diet fed mice at age 7–9 weeks. For other diets, 7–10 weeks old wild-type and Marc1−/− mice were fed with HFHFD or CDAA-HFD for 12 weeks, respectively. At the end of experiments mice were sacrificed, liver and serum collected and indicated measurements performed as described in Methods. All mice were sacrificed at ad lib feeding state (non-fasted). Each group contains 10–11 mice. Mean ± s.e.m. are shown for all measurements. NA–not measured. (TIF) [file pgen.1011179.s016.tif]

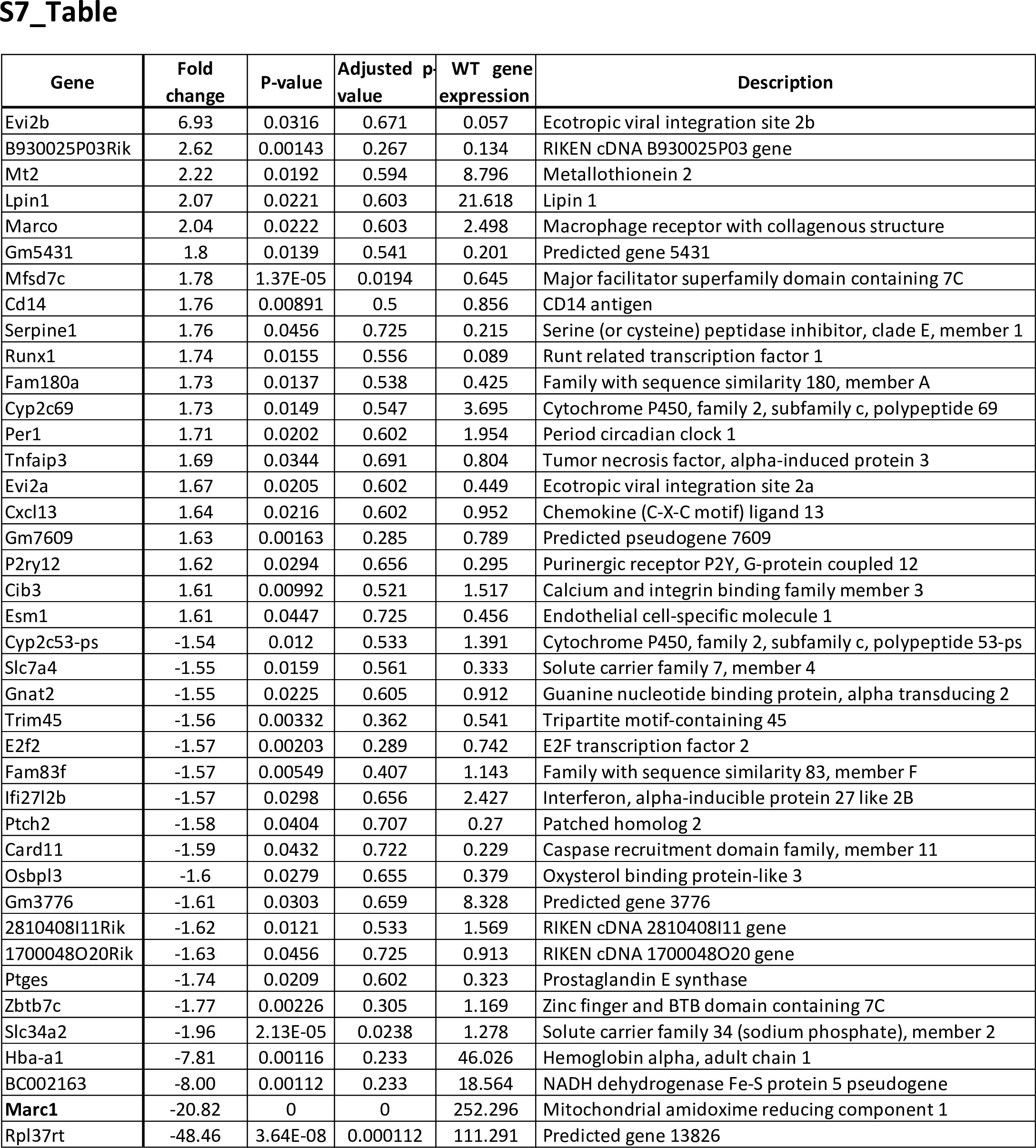

Supplement: S7 Table — RNAseq was performed using livers from ad lib chow fed WT and Marc1 KO male mice (n = 8–9). Fold changes >1.5X between Marc1 KO vs WT mice are shown. P-value calculated by t-test, adjusted p-values corrected by multiple variants. WT gene expression–mean values of mRNA expression (TPM) of wild type mice. (TIF) [file pgen.1011179.s017.tif]

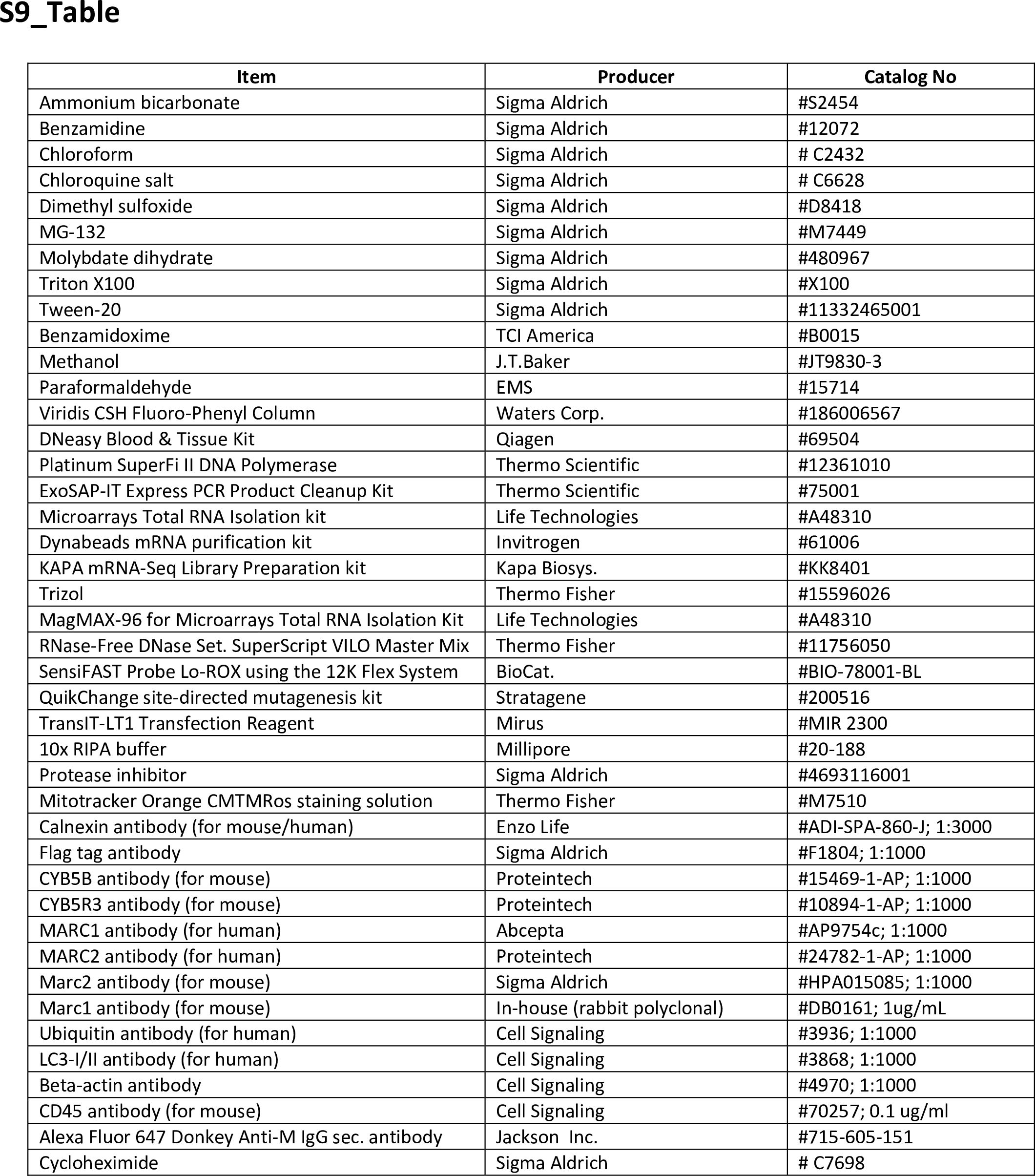

Supplement: S9 Table — (TIF) [file pgen.1011179.s019.tif]
