# Supplementary material for: Divergent role of Mitochondrial Amidoxime Reducing Component 1 (MARC1) in human and mouse
Source: PLoS Genet. 2024 Mar 4;20(3):e1011179. doi: 10.1371/journal.pgen.1011179 (PMC10939284; doi:10.1371/journal.pgen.1011179)
Supplement: Attachment — Submitted filename: Response to reviewers_Smagris_Marc1.docx [file pgen.1011179.s021.docx]

Dear Dr. Lin and Dr. Tang

I hope this message finds you well. I am writing to express my gratitude for the time and effort you have dedicated to reviewing our scientific paper titled “Divergent role of Mitochondrial Amidoxime Reducing Component 1 (MARC1) in human and mouse".

We would like to thank reviewers for the thoughtful review of our manuscript and the interest in our work. We appreciate their constructive feedback that helped us to improve the manuscript. Below, please find the response to the reviewers’ comments (in blue font). We are also submitting both a marked-up copy of the original manuscript showing all the changes that have been made, and the clean version of the revised manuscript.

Response to Reviewer #1:

Smagris and colleagues have examined the linkage between variations in MARC1 and its protein stability and have then gone on to evaluate the effects of MARC1 deficiency in mice. Variation in the gene encoding MARC1 have been linked to hepatic steatosis in people but a mechanistic understanding of how altered MARC1 activity affects liver lipid content is lacking. This work is therefore regarded as of high importance. The studies are well done and the experimental progression is very logical. There were a few remaining questions that are fairly minor.

As proof of concept, it would be useful to complement the Marc1/2 KO hepatocytes by expressing the human and mouse MARC1 protein to see if there are species differences in enzyme activity.

We agree that this could be an informative experiment. Moreover, we have previously considered the possibility of conducting such an experiment. However, the results of this experiment could be hard to interpret due to multiple considerations. Specifically: 1) the experiment design will require repeated AAV8 transduction in Cas9 transgenic mice – to silence mouse Marc1/2 expression (by Marc1/2 gRNA delivery in AAV8) in liver, and then delivery of the second AAV8 with human or mouse MARC1 gene 2-3 weeks later. It was reported that repeated AAV8 administration leads to very significant reduction of second AAV8 delivery in mouse liver due to generation of a very strong neutralizing antibody response (Choi et al., J Med 2023 PMID: 36994804). We also considered to use both viruses together to inject at the same time (mouse gene knockout plus human gene overexpression) – but this way we could face unpredictable AAV8 expression in liver as both viruses will compete for the same transduction pathways in the same cell; 2) AAV8-mediated expression of human and mouse MARC1 in mouse hepatocytes may result in hyper-physiological levels of the protein (compared to endogenous mouse Marc1 protein expression). This could lead to an inadequate amount of MARC1 cofactors’ CYB5R3 and CYB5B (MARC1 works in complex with CYB5R3 and CYB5B on the surface of mitochondria), as well as there is a potential risk of incompatibility between human MARC1 and mouse Cyb5R3 and Cyb5b proteins. After careful consideration of a questionable physiological relevance of the study outcomes, we decided to do not proceed with this experiment.

To distinguish between impaired stability and translation of the MARC1 variant protein, studies with cycloheximide would be useful.

We agree with the reviewer’s suggestion, conducted the study and added a figure to the Supplemental data (currently figure S6). After determining the optimal dose and dosing time points of cycloheximide (CHX) for Huh-7 cells, we compared MARC1 WT and p.165T protein variant stability changes after CHX treatment. CHX treatment (300 μg/ml) led to significant reduction in both WT and p.165T MARC1 protein levels after 3, 6 and 9 hours of treatment compared to control group (DMSO, S6 Fig. C). In the same experiment, combination of CHX with proteasomal (MG-132) or lysosomal (chloroquine) inhibitors led to protection of WT MARC1 protein from degradation. However, addition of proteasomal (MG-132) or lysosomal (chloroquine) inhibitors to CHX for cells expressing MARC1 p.165T variant did not lead to p.165T variant protein protection from degradation compared to CHX alone treatment (S6 Fig. C and D). In conclusion, these data indicate that MARC1 p.165T variant instability is not caused by either proteasomal or lysosomal degradation.

What is the expression pattern of MARC2 in humans?

Currently, we do not possess in-house human tissue expression database, but commonly used publicly available online database GTExportal (<https://gtexportal.org/home/gene/MARC2>) shows highest MARC2 mRNA expression in liver and thyroid with lower expression in other organs. We added it to the text in the Results section under “Marc1 is predominantly expressed in the liver in mice” to address this question.

Last paragraph of the results: Mice don’t have “genders”. This is a human-specific concept. This should also be fixed in the methods.

We agree with the remark and replaced “genders” with “sexes” in the manuscript and the method section per reviewer’s suggestion.

Response to Reviewer #2:

In their exome-wide association study, Eriks et al uncovered several novel loss of function variants in MARC1. They conducted studies to elucidate the protein stability and localization of well-known A165T and M187K mutations. Their finding revealed that the A165T mutation results in lower protein stability compared to the wild type. They also generated the Marc1 deficient mice which exhibited no significant differences in NASH progression. The generated Marc2 mice displayed neurological behavior defects. They also found Marc2 as a key enzyme involved in N-hydroxylated amidine reduction in mouse liver.

Despite the presence of known data and numerous negative findings, the creation of Marc1 knockout mice and the development of a specific Marc1 antibody hold promise for advancing the field. The authors propose that their study serves as an example of the non-consistency between human and mouse models, emphasizing the importance of investigating such discrepancies in research outcome.

1: In Figure 8, the AAV-mediated knockdown of Marc1 and Marc2 was exclusively conducted on hepatocytes. It would be valuable to know if these knockdown mice were subjected to a NASH-inducing diet. Additionally, exploring the effects of overexpressing the A165T and M187K mutants in Marc1 knockdown mouse livers could provide interesting insights.

For the experiment in Figure 8 we used primary hepatocytes from Cas9-ready transgenic mice with AAV8-mediated knockdown of Marc1, Marc2 and Marc1/2 on regular chow diet. We have previously subjected mice with Cas9 mediated gene deletion of Marc1, Marc2 and Marc1/2 to NASH-inducing diet (high fat high fructose diet, Research Diets, Cat. No D09100310) for 12 weeks in a parallel experiment. Unfortunately, we observed only partial deletion of Marc1, Marc2 and Marc1/2 at the end of this study (gene deletion in this model depends on time after AAV administration and the diet; in general, in our experience, it is common to observe reduced knockdown efficiency on high fat diet). Thus, we decided to run a shorter study (2-4 weeks) on regular chow diet for the enzymatic activity experiment in primary hepatocytes that resulted in efficient knockdown of Marc1, Marc2 and Marc1/2 (Figure 8A).

As for exploring the effects of overexpression of the A165T and M187K mutants in Marc1 knockdown mouse livers, we agree that it could provide an interesting insight and we indeed considered to conduct such an experiment earlier. However, this experiment may lead to non-physiologically relevant results due to multiple reasons. Specifically: 1) the experiment design will require repeated AAV8 transduction in Cas9 transgenic mice – to silence mouse Marc1/2 expression (by Marc1/2 gRNA delivery in AAV8) in liver, and then delivery of the second AAV8 with human MARC1 gene (WT, A165T, M187K) 2-3 weeks later. It was reported that repeated AAV8 administration leads to very significant reduction of second AAV8 delivery in mouse liver due to generation of a very strong neutralizing antibody response (Choi et al., J Med 2023 PMID: 36994804). We also considered to use both viruses together to inject the constructs at the same time (mouse gene knockout plus human gene overexpression) – but this way we could face unpredictable AAV8 expression in liver as both viruses will compete for the same transduction pathways in the same cell; 2) In addition, AAV8-mediated expression of human MARC1 in mouse hepatocytes will lead to hyperphysiological levels of the protein (compared to endogenous mouse Marc1 protein expression), and there will be a difference between the expression levels of WT, A165T and M187K, making it hard to compare. This could lead to an inadequate amount of MARC1 cofactors’ CYB5R3 and CYB5B (MARC1 works in complex with CYB5R3 and CYB5B on the surface of mitochondria), as well as there is a potential risk of incompatibility between human MARC1 and mouse Cyb5R3 and Cyb5b proteins. After careful consideration of a questionable physiological relevance of the study outcomes, we decided to do not proceed with this experiment.

2: Have the authors investigated the impact of a normal high-fat diet (HFD) on Marc1-deficient mice

We performed such experiment in male Marc1-deficient mice and the data is summarized in manuscript in S5 Table. After 11 weeks of feeding of Marc1 KO mice with high fat diet (Research Diets, Cat. No D12492; 60% fat by calories) we observed plasma ALT and hepatic cholesterol ester reduction in Marc1 KO mice, but additional metabolic phenotyping did not reveal any changes in body mass, liver triglycerides or glucose metabolism.

3: In Figure S4, the protein levels for A165T are noticeably higher in MG-132 and Chloroquine compared to DMSO. This raises questions about the authors' claim that the A165T variant was not rescued by these inhibitors. Is there any existing literature reporting the degradation of wild-type Marc1 through the proteasomal or lysosomal pathways. Further investigation into protein degradation using CHX treatment will be interesting.

The original Figure S4 is now Figure S6 in the revised manuscript. We agree that in S6 Figure MARC1 p. A165T variant protein total amount indeed looks higher after MG-132 and Chloroquine treatment compared to the control group (DMSO), however still much lower than the WT protein expression level. We performed MARC1 and Calnexin (protein loading control) Western Blot signal densitometry for the experiment shown in Figure S6 and calculated the ratio between MARC1 and Calnexin signal. We observed very minimal nonsignificant increase of MARC1 A165T/Calnexin signal ratio only after MG-132 treatment (but not after Chloroquine treatment) and this ratio was proportional to WT MARC1/Calnexin signal changes after MG-132 treatment which also showed small increase. We repeated this experiment once for Chloroquine and twice with MG-132 with the same results. We currently added the densitometry graph as an additional panel to Figure S6.

As far as we are aware, there are no published data on MG-132 and Chloroquine rescue of MARC1 WT or p.A165T protein variant.

We conducted further the investigation of protein degradation using CHX treatment per reviewer suggestion and added the data from this experiment to Figure S6. After determining the optimal dose and dosing time points of cycloheximide (CHX) for Huh-7 cells, we compared MARC1 WT and p.165T protein variant stability changes after CHX treatment. CHX treatment (300 μg/ml) led to significant reduction in both WT and p.165T MARC1 protein levels after 3, 6 and 9 hours of treatment compared to control group (DMSO, S6 Fig. C). In the same experiment, combination of CHX with proteasomal (MG-132) or lysosomal (chloroquine) inhibitors led to protection of WT MARC1 protein from degradation. However, addition of proteasomal (MG-132) or lysosomal (chloroquine) inhibitors to CHX for cells expressing MARC1 p.165T variant did not lead to p.165T variant protein protection from degradation compared to CHX alone treatment (S6 Fig. C and D). In conclusion, these data indicate that MARC1 p.165T variant instability is not caused by either proteasomal or lysosomal degradation.

4: The image quality in Figure 3 is suboptimal, particularly in discerning whether wild-type MARC1 localizes with mitochondria.

We repeated this experiment one more time and had the same results: WT MARC1 protein always colocalizes with mitochondria the same way as in manuscript Fig. 3. One of reason for the not ideal matching of MARC1 WT with mitochondria could be that MitoTracker (Orange CMTMRos) mitochondria marker accumulates in active mitochondria matrix by covalently binding the thiol groups of mitochondrial proteins, while the majority of MARC1 protein except the small transmembrane part is located on cytosolic surface of mitochondria. We also cannot rule out minimal colocalization artifacts from MARC1 protein overexpression (human MARC1 gene was expressed under CMV promoter), were part of new made MARC1 protein is still migrating from ER to mitochondria. We understand the limitation of showing only a single panel for each condition, thus we replaced Fig. 3 data with the second experiment data, where we can better see MARC1 WT protein colocalization with mitochondria. We also added the data from the original Fig. 3 experiment to the supplemental figure file as S5 Figure to strengthen the findings.

5: In Figure 4A-B, the statement "we found Marc2 mRNA expression was almost two times higher than Marc1 in mouse liver" may be challenging to interpret due to the inherent difficulties in comparing expression levels between two different genes.

We agree with the reviewer and removed this sentence from the manuscript.
